# Genomic profiling and HER2-targeted therapy: a case series of Chinese salivary duct carcinoma patients and its clinical implications

**DOI:** 10.3389/fonc.2026.1727362

**Published:** 2026-02-25

**Authors:** Xiang Huang, Wei Yu, Xiaomo Li, Boning Cai, Baolin Qu, Jing Chen, Pei Zhang, Xinrui Chen, Yanli Liu, Shengqiang Feng, Lin Gui, Fang Liu

**Affiliations:** 1 Department of Radiation Oncology, Senior Department of Oncology, the First Medical Center of PLA General Hospital, Beijing, China; 2 Genetron Health (Beijing) Technology, Co. Ltd, Beijing, China; 3 Department of Medical Oncology, National Cancer Center/National Clinical Research Center for Cancer/Cancer Hospital, Chinese Academy of Medical Sciences & Peking Union Medical College, Beijing Key Laboratory of Clinical Study on Anticancer Molecular Targeted Drugs, Beijing, China; 4 Health Service Department of the Guard Bureau, The Joint Staff Department, Beijing, China

**Keywords:** driver mutation, HER2-targeted therapy, pertuzumab, pyrotinib, salivary duct carcinoma, trastuzumab

## Abstract

**Purpose:**

Salivary duct carcinoma (SDC) is a rare and aggressive subtype of salivary gland cancers (SGCs). Currently, there is no approved targeted therapy for this devastating disease. This study aimed to identify actionable genomic alterations in Chinese patients with SDC and explore the clinical efficacy of human epidermal growth factor receptor 2 (HER2)-targeted therapies in this population.

**Methods:**

Somatic and genomic DNA were isolated from SDC samples and the corresponding peripheral blood controls, respectively. To explore the potential of targeted therapy, next-generation sequencing (NGS) was used to identify genomic alterations, especially mutations with clinical significance.

**Results:**

The most prevalent driver mutations in this SDC cohort are *TP53*, *HER2*, *PIK3CA*, *HRAS*, and *NF1*. Except for *TP53*, the other four driver mutations are targetable. We identified three novel *HER2* mutations (D769H, H878Y, and an exon 16 skipping mutation) in SDC, marking the first report of these mutations in this cancer type. Two metastatic SDC patients with *HER2* amplification responded to HER2 tyrosine kinase inhibitor (TKI) pyrotinib and the combination of trastuzumab and pertuzumab, respectively.

**Conclusion:**

Our work underscores the potential of genomic profiling to guide precision therapy in SDC, with HER2-targeted treatments offering promising therapeutic avenues.

## Introduction

Salivary gland malignancies (SGMs) are a group of rare tumors, representing approximately 5% of all head and neck cancers (1). SGMs constitute more than 20 histological subtypes, including mucoepidermoid carcinoma (MEC), adenoid cystic carcinoma (ACC), acinic cell carcinoma (AciCC), secretory carcinoma (SC), and SDC (2). As the most aggressive subtype of all SGM subtypes, SDC accounts for about 5% of SGM cases (3, 4). 54-68% of SDC patients had lymph node involvement at presentation, resulting in a poor prognosis (5–7). For instance, one National Cancer Data Base study showed that SDC patients with nodal metastasis (N+) had significantly worse overall survival (OS) than those without (N0) (5-year OS, 35% vs. 73%; P < 0.001) (5).

Given the rarity of SDC, the guidelines for SDC management are limited (1, 8). Primary treatments for localized SDC include surgery and chemoradiotherapy. In the case of recurrent or metastatic (R/M) SDC, there is no approved therapy, and systemic treatment is challenging. Of note, more than 90% of SDC tumors express androgen receptor (AR), and 29-43% of SDC tumors overexpress HER2 (7, 9). AR and HER2 are well-established therapeutic targets in multiple cancer types, including prostate and breast cancer (10, 11). Recently, the ESMO-EURACAN guideline recommended confirmation of AR/HER2 expression status for all SDC patients (8). Moreover, the ASCO SGM guideline gave a moderate recommendation of targeted therapy for AR/HER2-positive SDC patients if clinical trials are not available (1). In addition to *AR* and *HER2* overexpression, actionable genomic alterations, including *BRAF*, *HER2*, *PIK3CA* mutations, and *RET*-fusion genes have been detected in SDC (12, 13). These results indicate that genomic profiling may offer SDC patients opportunities for precision therapy and clinical trials.

The histology of SDC resembles high-grade invasive duct carcinoma of the breast (3). Historically, HER2-positive breast cancer (BC) was a very aggressive histology with a poor prognosis (14). However, HER2-targeted therapy drugs, including monoclonal antibodies, TKIs, and antibody-drug conjugates (ADCs), have significantly improved the outcomes of patients with HER2-positive BC and become the standard of care for this disease (15). Recently, HER2 monoclonal antibodies and ADC have shown promising antitumor activity in HER2-positive SGC. In two single-arm, open-label, phase 2 trials of HER2-positive R/M SDC, patients were treated with docetaxel and HER2 monoclonal antibody trastuzumab, which resulted in objective response rates (ORRs) of 70% and 80%, respectively (16, 17). Similarly, in a phase 2 basket trial of multiple histologies, HER2 ADC ado-trastuzumab emtansine achieved an ORR of 90% in 10 *HER2*-amplified SDC patients, including five complete responses (CR) (18).

Here, we describe a case series of R/M HER2-positive SDC patients treated with late-line HER2-targeted therapy. One patient showed a partial response to HER2 TKI pyrotinib, and another responded to the combination of trastuzumab and pertuzumab after disease progression on pyrotinib. Additionally, we provide a preliminary analysis of driver and targetable mutations in 13 SDC patients. To our knowledge, this is the first study to profile the genomic landscape of SDC in Chinese patients.

## Materials and methods

**Figure 1:**
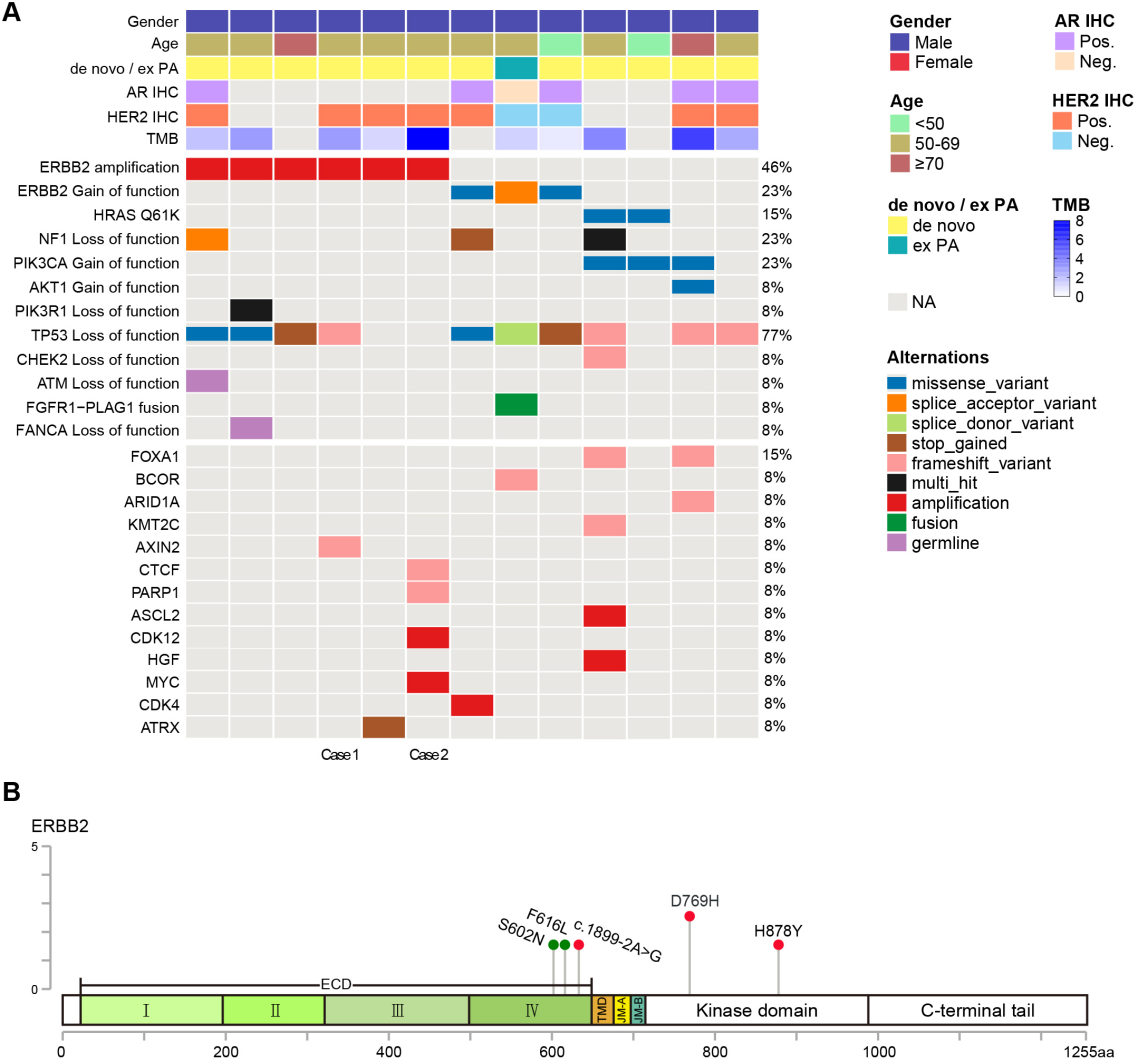
The genetic landscape of salivary duct carcinoma (SDC). **(A)** Clinical data (top) and selected genetic alterations (bottom) in 13 Chinese SDC patients. **(B)** Locations of *HER2* activating mutations in this Chinese SDC cohort. Activating mutations and variants of unknown significance (VUSs) were marked in red and green, respectively.

A total of 13 patients with newly diagnosed salivary duct carcinoma from March 2017 to October 2022 were included in this retrospective study. Inclusion criteria comprised: subjects with a primary pathological/clinical diagnosis of SDC, availability of tumor tissue samples, and matched peripheral blood samples. Exclusion criteria comprised: insufficient sample quantity or quality that did not meet the experimental requirements; incomplete clinical data, including basic demographic information and clinical examination/diagnostic results; or failure to complete the NGS testing due to instrumental or human factors. Formalin‐fixed paraffin‐embedded (FFPE) tissue and matched peripheral blood samples were collected for somatic and germline DNA extraction, respectively. Demographic data of patients were summarized in **Figure 1A**. The study protocol was approved by the Ethical Committee of the Chinese People’s Liberation Army (PLA) General Hospital, and the participants gave written informed consent before sample collection. Hybridization capture-based targeted NGS with an 831-gene panel (Onco Panscan™, Genetron Health) was performed on the Illumina NovoSeq 6000 platform (Illumina), which covers somatic single-nucleotide variants (SNV), insertions/deletions (indels), copy number variations, structural variations, and germline SNVs/indels. DNA extraction, library preparation, DNA sequencing, and variant classification were performed as described (19). A minimum allele frequency of 10% and a median sequencing depth of at least 500× were used as quality thresholds. *HER2* amplifications were called when >4-fold change was detected on normalized depth or further ddPCR proved amplification with a fold change of 2.6-4. Somatic variants annotated as “oncogenic” or “likely oncogenic” in the OncoKB database (https://www.oncokb.org/) were considered variants of clinical significance and used for further analysis. PD-L1 expression was examined with the PD-L1 IHC 22C3 pharmDx assay (Agilent Technologies, Carpinteria, CA, USA).

## Results

### Genomic profile of Chinese SDC patients

The most frequently mutated gene in this Chinese SDC cohort was *TP53* (77%). Genomic alternations in the receptor tyrosine kinase (RTK)/MAPK pathway (85%, 11/13) include *HER2* amplification (n = 6), *HER2* gain-of-function mutations (n = 3), *HRAS* Q61K (n = 2), and loss-of-function mutations in *NF1* (n = 3). Genomic alterations in the PI3K pathway genes (31%, 4/13) include activating mutations in *PIK3CA* (n = 3), *AKT1* (n = 1), and inactivating mutations in *PIK3R1* (n = 1). The list of all mutations of clinical significance found in our cohort is shown in **Figure 1A**. Among eight tumors with PD-L1 expression data, four were PD-L1 positive, and one had a TPS > 50%. Additionally, all 13 SDC tumors had low tumor mutational burden (TMB).

In this Chinese SDC cohort, we found three activating *HER2* mutations (D769H, H878Y, and c.1899-2A>G) (**Figure 1B**), which were mutually exclusive with *HER2* amplification. The splicing site variant c.1899-2A>G is a likely oncogenic *HER2* exon 16 skipping (*HER2*Δex16) mutation (20). The diverse, potentially actionable genomic alterations identified in this SDC cohort underscored disease heterogeneity and provided a rationale for further investigation in molecularly informed clinical trials.

## Case presentation

### Case 1

A 62-year-old male presented with a left parotid gland mass and facial anesthesia in 2018. Contrast-enhanced computed tomography (CT) revealed a 3.1 cm-diameter nodule in the left parotid gland and multiple lymph node metastases in the left neck. He underwent surgical resection of the parotid mass and deep/superficial lobe of the left parotid gland, as well as left neck lymph node dissection on May 25^th^, 2018. Postoperative histopathological examination confirmed a 4.5×2.5×2.5 cm, group stage IVA (pT3N2bM0) SDC, with invasion of nerve and surrounding striated muscle tissue, and multiple metastatic lymph nodes. He further underwent left facial nerve resection and left radical neck lymphadenectomy on June 8^th^, 2018, and vascular cancer emboli were found. The patient received concurrent chemoradiotherapy from July 11^th^ to August 21^st^, 2018.

A follow-up chest CT scan in December 2019 revealed multiple nodules with poor blood perfusion, suggesting liver metastases. The patient received six cycles of docetaxel, lobaplatin, and nimotuzumab and achieved a partial response. Treatment response was assessed by Response Evaluation Criteria in Solid Tumors (RECIST) criteria. In June 2019, new brain and liver metastases indicated disease progression. Treatment was then changed to gemcitabine, S-1, and anlotinib. Unfortunately, accelerated progression of liver metastases and liver function damage (AST 111.7 U/L, ALT 115.6 U/L) were observed on July 31^st^, 2019.

**Figure 2:**
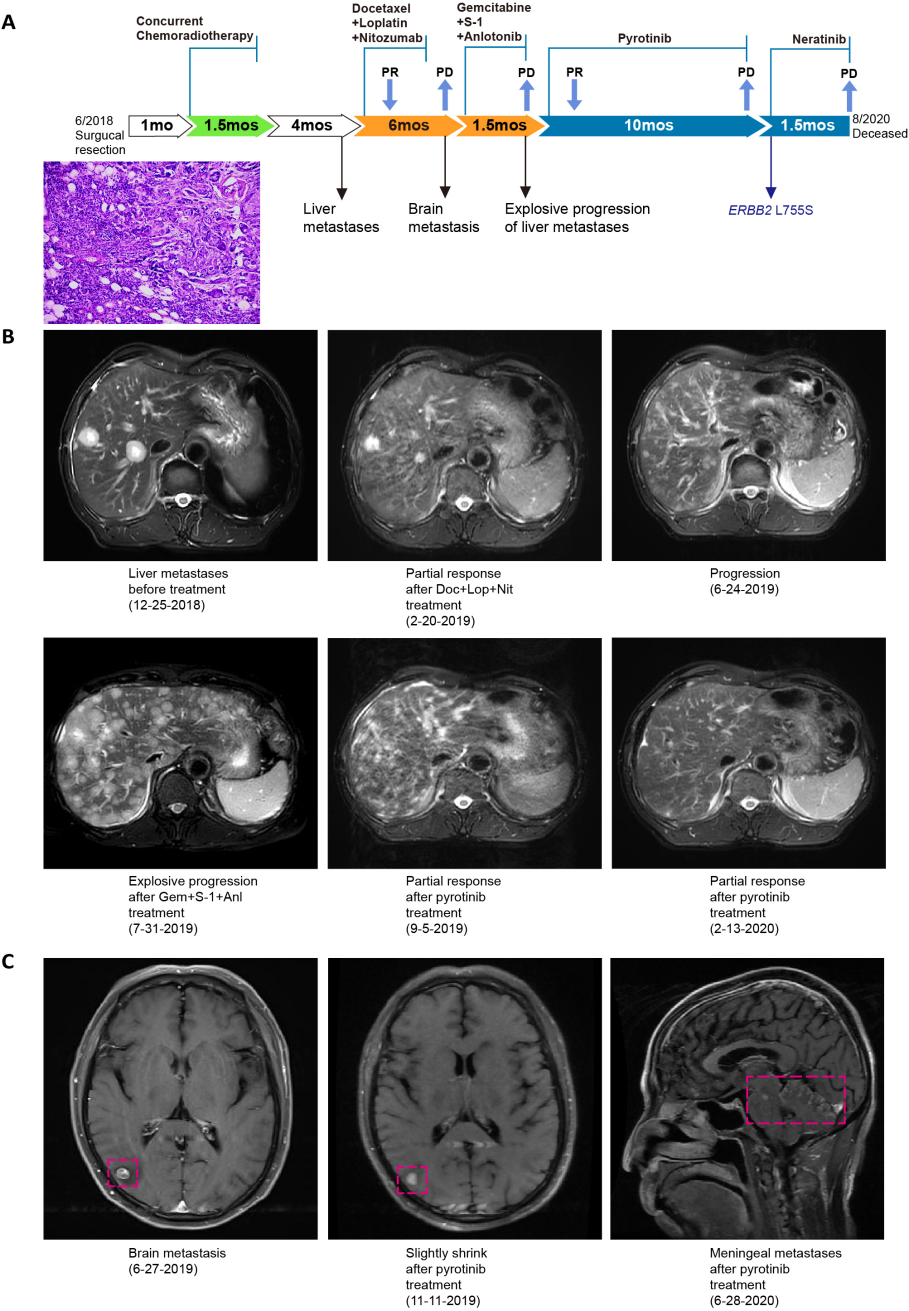
*HER2* amplification-positive SDC case 1 summary. **(A)** Summary of disease course and treatment procedure. PR, partial response; PD, progressive disease; mo, months. A H&E-stained slice of tumor tissue, magnified 100 times, was shown. Representative images showing liver metastases **(B)** and brain metastasis **(C)** before and after treatment with HER2 TKI pyrotinib.

The failure of chemotherapy led us to explore the possibility of targeted therapy. Genetic testing of the resected tumor tissue with a multi-gene NGS panel revealed *HER2* amplification (fold change, 5.3). The patient was treated with HER2 TKI pyrotinib (320 mg, QD) from August 3^rd^, 2019. After one month, a partial response of liver metastases and a slight shrinkage of brain metastases were observed. The liver function returned to normal (AST 8.3 U/L, ALT 25.4 U/L) after active liver protection management. Diarrhea (grade 2) was the only adverse event during pyrotinib treatment. In June 2020, the patient experienced disease progression, including vomitus, dizziness, and new meningeal metastases. Genetic profiling of his plasma ctDNA showed the presence of *HER2* amplification (fold change, 2.61) and *HER2* L755S mutation with a VAF of 13.43% (**Figure 2**). Preclinical studies showed that the HER2 L755S mutant produced resistance to trastuzumab and the HER2 kinase inhibitor lapatinib, but was sensitive to another HER2 kinase inhibitor, neratinib (21, 22). In a global, multicenter basket study of solid tumors with *HER2*/*HER3* mutations, neratinib achieved a RECIST response in a lung cancer patient with the *HER2* L755S mutation (23). With the informed consent from the patient, he started off-label treatment of neratinib (240 mg, QD). Unfortunately, the patient died two months later due to progressive meningeal metastases and pulmonary infection. This *HER2*-amplification SDC case, together with another case report of the response to pyrotinib/bicalutamide in a patient with HER2/AR-positive, metastatic SDC (24), indicated that HER2 TKI may be a potential treatment option for SDC patients with *HER2* alterations.

### Case 2

In March 2017, a 54-year-old Chinese man was admitted to our hospital due to a spherical mass in the right parotid gland area revealed by ultrasonography during routine physical examination, accompanied by blurred vision in the right eye and weakness in closing eyelids. He underwent right parotidectomy, facial nerve dissection, and right neck radical lymphadenectomy on May 12^th^, 2017. Postoperative histopathological examination confirmed a 2.5×2.5×2cm poorly differentiated carcinoma of salivary gland origin, with lymph node metastases (pT2N2M0, stage IVA). The patient received concurrent chemoradiotherapy from June to October 2017.

**Figure 3:**
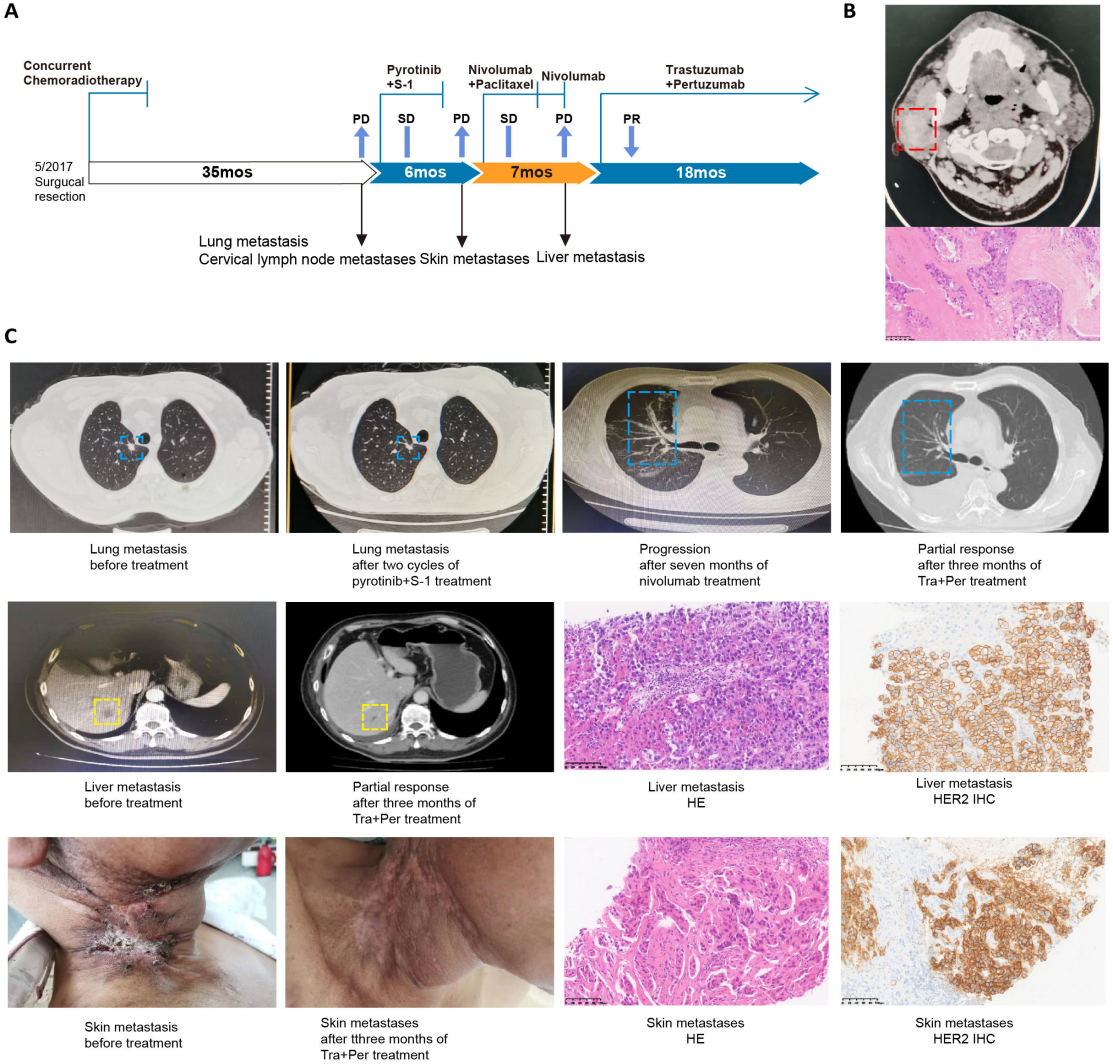
*HER2* amplification-positive SDC case 2 summary. **(A)** Summary of disease course and treatment procedure. PR, partial response; PD, progressive disease; mo, months. **(B)** Computed tomography image and H&E staining of the primary SDC tumor magnified 100 times. **(C)** Images showing multiple lesions in the patient’s lung, liver, and neck skin. A partial response was observed after HER2-targeted therapy with trastuzumab plus pertuzumab. H&E and HER2 staining, magnified 100 times, of the neck skin and liver lesions were included as controls.

In March 2020, the patient reported scattered itching masses in the neck. PET-CT imaging revealed nodules in the upper lobe of the right lung, as well as hypermetabolic lymph nodes in the right posterior cervical space, left neck, and clavicular region, indicating metastases. Genetic testing of the FFPE primary tumor tissues with a multi-gene panel NGS revealed a TMB of 8 mutations per megabase (mut/Mb), microsatellite stable (MSS), and *HER2* amplification (fold change, 9.4) (**Figure 3**). His treatment was changed to pyrotinib plus S-1 in May 2020. He tolerated the combination regimen well, and a regular review of chest CT in a local hospital indicated that the lung lesion was stable.

In November 2020, the patient reported neck skin mass enlargement, upper limb pain, and fingertip numbness. The histopathological examination of the skin mass biopsy suggested metastatic carcinoma of salivary gland origin. Immunohistochemical (IHC) staining indicated HER2 (1+). Treatment was changed to nivolumab plus paclitaxel for six cycles with stable disease (pulmonary lesion contracted). The patient continued on maintenance nivolumab. In July 2021, disease progression was noted, including new enlargement of metastases and subcutaneous nodules in the right neck. Histopathological analysis of liver and neck lesion biopsies suggested infiltration of poorly differentiated carcinoma with salivary gland origin. The HER2 IHC status of liver and neck lesion biopsies was 2+ and 3+, respectively. The patient started an anti-HER2 targeted therapy regimen of trastuzumab (Initial dose 8mg/kg, thereafter 6mg/kg, Q3W) and pertuzumab (Initial dose 840mg, thereafter 420mg, Q3W) in September 2021. After two cycles, the neck, liver, and lung lesions shrank and completely regressed, and this dramatic response lasted for 16 months. Our case series, together with the recent approval of trastuzumab deruxtecan in HER2-positive solid tumors (25), support the use of HER2-targeted therapies in HER2-positive SDC treatment.

## Discussion

SDC is one of the most aggressive subtypes of SGCs, and patients with this disease have a poor prognosis (1, 4). An analysis of 495 SDC patients in the National Cancer Database revealed that 52% had locally advanced disease, and 8% had metastatic disease (26). The 5-year OS for these two subgroups is 40% and 0%, respectively. Furthermore, adjuvant radiotherapy or chemotherapy does not affect patient survival. Therefore, there is an urgent need to develop effective systemic therapy for SDC.

The identification of targetable mutations provided cancer patients with the opportunity for precision therapy and clinical trials. Due to the rarity of SDC, there is limited data on targetable genomic alterations in this disease. The genomic profiling study of an American SDC cohort (n = 41) revealed that the most common GAs are *TP53* (53.7%), *PIK3CA* (36.6%), *HER2* (26.8%), *HRAS* (26.8%) (12). Consistently, the most common GAs in a European SDC cohort (n = 66) are *TP53* (51%), *PIK3CA* (32%), *HER2* (27%), and *HRAS* (22%) (27). To our best knowledge, our work is the first SDC genomic profiling study in the Chinese population. Compared to the American and European SDC cohorts, the Chinese SDC cohort had a similar prevalence of *TP53* (77%), *PIK3CA* (23%), and *HRAS* (15%) mutations, but a higher prevalence of *HER2* alterations (69%) and *NF1* (23%) mutations (12, 27). *HER2*, *PIK3CA*, *HRAS*, and *NF1* mutations are targetable.

*HER2* alterations, including amplification, overexpression, and point mutations, are common driver mutations in multiple cancer types (11). A broad spectrum of anti-HER2 agents, including monoclonal antibodies, TKIs, and ADCs, has been approved for the treatment of HER2-positive BC (11). Approximately 26%–38% of SDC tumors are HER2-positive (7, 9, 28, 29). The high prevalence of HER2-positive tumors in SDC makes it an attractive target for precision cancer therapy. In a prospective phase 2 trial for HER2-positive SDC (n = 57), trastuzumab plus docetaxel achieved an ORR of 70.2% (16). Median progression-free survival (PFS) and OS are 8.9 and 39.7 months, respectively. In the phase 2 MyPathway basket trial, trastuzumab plus pertuzumab achieved an ORR of 60% (1 complete response, 8 partial responses) in 15 patients with HER2-positive SGC (30). Consistently, we observed that one patient with HER2-positive SDC responded to the trastuzumab/pertuzumab doublet regimen after disease progression on chemotherapy and HER2 TKI pyrotinib. Results of the DESTINY-PanTumor02 trial showed that trastuzumab deruxtecan (T-DXd) had an overall ORR of 37.1% in 267 patients with non-NSCLC/non-CRC HER2-expressing (IHC 3+/2+) solid tumors (31). Among 19 patients with SGC in this cohort, trastuzumab deruxtecan achieved an ORR of 42.1% (8/19). Based on the results of this trial and the other two trials (DESTINY-Lung01 and DESTINY-CRC02), the FDA granted accelerated approval of trastuzumab deruxtecan for unresectable or metastatic HER2-positive (IHC 3+) solid tumors (25). Consistently, a pooled analysis of two phase 1 trials showed that trastuzumab deruxtecan achieved an ORR of 58.8% in 17 HER2-expressing SGC patients, including 9 SDC cases (32).

Little is known about activating *HER2* mutation in SDC. In this Chinese SDC cohort, we found three *HER2* tyrosine kinase domain (TKD) activating mutations (D769H, L755S, and H878Y) and an exon 16 skipping variant (c.1899-2A>G) (21, 33). To our best knowledge, this is the first report of these *HER2* mutations in SDC. The rare and oncogenic *HER2* Δex16 mutations have a pan-cancer prevalence of 0.05% in the Chinese population (18/38,680 and 22/40,996) (20, 34). Deletion of exon 16 resulted in an in-frame deletion in the transmembrane domain of HER2, which led to the formation of constitutively active *HER2* homodimers (35). Recently, the FDA approved two HER2 kinase inhibitors, sevabertinib and zongertinib, for the treatment of NSCLC patients with HER2 tyrosine kinase domain (TKD) activating mutations (36, 37). Three ongoing clinical trials aimed to evaluate the efficacy of sevabertinib and zongertinib in solid tumors harboring HER2 activating mutations (NCT06760819, NCT06581432, NCT06324357). Future large multi-center clinical trials are required to determine the optimal HER2-targeted therapy for SDC patients with *HER2* overexpression or activating mutations.

A significant portion of cancer patients treated with targeted therapy will eventually develop resistance. The most common *HER2* mutation in BC, L755P/S, is known to cause resistance to HER2 antibody trastuzumab and HER2 TKI lapatinib (21, 38). In this study, one patient with *HER2*-amplified SDC responded to HER2 TKI pyrotinib, but his disease progressed subsequently. A liquid NGS test after disease progression revealed an HER2 L755S kinase domain mutation, which could mediate resistance to pyrotinib. Preclinical studies showed that the L755S mutant was sensitive to HER2 TKI poziotinib, and partially sensitive to neratinib (21, 38, 39). Future clinical studies are needed to develop strategies to overcome HER2-targeted therapy resistance mediated by L755X mutations.

HRAS is a novel therapeutic target in head and neck cancer (40). To activate downstream signaling, HRAS proteins must be localized to the plasma membrane, which requires a post-translational modification named farnesylation catalyzed by farnesyltransferases (41). Tipifarnib, a potent farnesyltransferase inhibitor (FTI), blocks HRAS signaling through displacing HRAS from the cell membrane. In a single-arm, open-label, phase 2 trial, tipifarnib achieved a durable ORR of 55% in 30 patients with R/M head and neck squamous cell carcinoma (HNSCC) (40). Based on this result, the FDA has granted a breakthrough therapy designation (BTD) to tipifarnib for the treatment of patients with *HRAS*-mutant, R/M HNSCC after disease progression on chemotherapy. In a phase 2 basket trial, 13 R/M SGC patients were treated with tipifarnib after progression on chemotherapy (42). One objective response was observed, and an additional five patients had >10% tumor regression. The median PFS and OS are 7 and 18 months, respectively.

The phosphatidylinositol 3-kinase (PI3K)-AKT pathway is one of the most activated pathways in various cancer types (43). Canonical activating mutations (E542K, E545K, and H1047R) in *PIK3CA* result in hyperactivation of PI3K signaling, which affects approximately 40% of HR-positive, HER2-negative BC patients. The FDA has approved PIK3CA inhibitor alpelisib in combination with fulvestrant for the treatment of *PIK3CA*-mutated, advanced, or metastatic BC and alpelisib monotherapy for *PIK3CA*-related overgrowth spectrum (PROS) (44, 45). *PIK3CA* mutations are common in SDC (46–48). In one SDC study, 21% of patients (7/34) had activating *PIK3CA* mutations, including E545K (3), E542K (2), and H1047R (3) (48). In this study, we observed three patients harboring activating *PIK3CA* mutations, including H1047R, E545Q, and N345K (49). To our best knowledge, this is the first report of *PIK3CA* E545Q/N345K mutations in SDC. Recently, the FDA approved the AKT inhibitor capivasertib with fulvestrant for hormone receptor (HR)-positive, HER2-negative locally advanced or metastatic BC with *PIK3CA, AKT1*, or *PTEN* alterations (50). Activating *PIK3CA* and *AKT1* mutations are common in metastatic castration-resistant prostate cancer (mCRPC) and often lead to resistance to chemotherapy (51). In a phase 1 trial of enzalutamide combined with capivasertib in patients with mCRPC, one patient who had a ≥ 30% PSA response at 4 weeks was found to have a *PIK3CA* I391M activating mutation (49, 52). In the phase 2 ProCAID trial for patients with mCRPC, adding capivasertib to chemotherapy prolonged median OS from 20.27 months to 31.15 months (53). Currently, the antitumor activity of capivasertib in mCRPC is under investigation in two phase 3 trials (CAPItello-280 and CAPItello-281). Interestingly, one metastatic SDC patient with concurrent HRAS Q61R/PIK3CA E545K mutations and high AR expression achieved an excellent response with the combination of alpelisib and bicalutamide (54). These results suggested that alpelisib and capivasertib may be potential treatment options for SDC patients with activating *PIK3CA* mutations.

In addition to SDC, molecular diagnostic approaches, including NGS and FISH, can help the diagnosis of other salivary gland tumor types (55). For instance, the detection of *CRTC1*-*MAML2* fusion gene by FISH can distinguish Warthin tumor and Warthin-like mucoepidermoid carcinoma (MEC) (56). The detection of *PRKD1* E710D mutation and *PRKD1/2/3* fusion genes can be used for the diagnosis of conventional and cribriform forms of polymorphous adenocarcinoma, respectively (57). Another common chromosomal alteration in salivary gland tumors is the rearrangement of the pleomorphic adenoma gene 1 (*PLAG1*) (58–60). It was originally discovered in pleomorphic adenoma, the most common subtype of SGC (58). The promoter swapping of *PLAG1* and *CTNNB1* results in abnormal activation of *PLAG1* expression in the salivary glands. Recent studies revealed that *PLAG1* rearrangements are prevalent in two aggressive subtypes of SGCs, myoepithelial carcinoma (MECA) and SDC (59, 60). In a MECA study, 53% (21/40) of patients harbored *PLAG1* rearrangements, including 7 *FGFR1*-*PLAG1* fusions (60). An analysis of 66 SDC patients revealed that 11 harbored *PLAG1* rearrangements, including one *FGFR1*-*PLAG1* fusion (59). In this SDC study, we also observed one patient harboring an *FGFR1*-*PLAG1* fusion.

## Conclusions

The findings of this study suggested that next-generation sequencing can provide useful information for the optimal treatment of SDC. Our results, together with previous genomic profiling studies, demonstrated that a significant portion of SDC patients harbored targetable mutations. These patients may consider participating in the basket trials of HER2, HRAS, and PIK3CA inhibitors. Our study highlights the potential of genomic profiling to revolutionize the management of SDC, with HER2-targeted therapies offering a promising avenue for improving patient outcomes in this aggressive cancer type.

## Data Availability

The original contributions presented in the study are included in the article/supplementary material. Further inquiries can be directed to the corresponding authors.

## Glossary

ACC: adenoid cystic carcinoma
AciCC: acinic cell carcinoma
ADCs: antibody-drug conjugates
AR: androgen receptor
BC: breast cancer
BTD: breakthrough therapy designation
CR: complete response
CT: computed tomography
FFPE: Formalin‐fixed paraffin‐embedded
HER2: human epidermal growth factor receptor 2
HNSCC: head and neck squamous cell carcinoma
H&E: hematoxylin and eosin
IHC: immunohistochemical
MEC: mucoepidermoid carcinoma
MSS: microsatellite stable
NGS: next-generation sequencing
ORRs: objective response rates
R/M: recurrent or metastatic
PI3K: phosphatidylinositol 3-kinase
RECIST: response evaluation criteria in solid tumors
RTK: receptor tyrosine kinase
SC: secretory carcinoma
SDC: salivary duct carcinoma
SGCs: salivary gland cancers
TMB: tumor mutational burden
TKI: tyrosine kinase inhibitor
TKD: tyrosine kinase domain
T-DXd: trastuzumab deruxtecan
VAF: variant allele frequency.
